# Characteristic of the acute drug intoxication conveyed by Kanagawa helicopter emergency medical service

**DOI:** 10.1002/ams2.255

**Published:** 2017-04-24

**Authors:** Mariko Sugita, Shigeo Higami, Shigeaki Inoue, Seiji Morita, Yoshihide Nakagawa, Sadaki Inokuchi

**Affiliations:** ^1^ Department of Emergency and Critical Care Medicine Tokai University Scool of Medicine Isehara Japan

**Keywords:** Aeromedical, air medical service, drug intoxication, helicopter, poisoning

## Abstract

**Aim:**

Various critical cases have been transported since the use of the Kanagawa Helicopter Emergency Medical Service (HEMS) started at Tokai University Hospital (Isehara, Japan) in 2002, including cases of acute poisoning. We analyzed the characteristics of acute poisoning cases conveyed by the HEMS.

**Methods:**

Kanagawa HEMS conveyed 3,814 cases from July 2002 to March 2013, and acute drug and poison intoxication was diagnosed in 131 of these cases. We undertook a descriptive statistical study of these cases.

**Results:**

The causative agent was found to be psychiatric prescription drugs in 39.7% of cases, pesticides in 29.7%, alcohol in 8.4%, analgesics in 5.3%, detergent or bleach in 6.1%, oil, natural gas, or thinner in 4.6%, and others in 6.1%. At HEMS contact, systolic blood pressure was less than 90 mmHg in 18.3% of cases, and 40.2% were in coma. Endotracheal intubation was carried out in 44.5% of cases, and 6.9% died within 24 h of hospital admission. The cases of poisoning that we transported in the HEMS were often in shock and/or coma on arrival at the field, and rapid endotracheal intubation was required in nearly half of them, as many were in a serious condition.

**Conclusion:**

We believe that outcomes were more likely to be improved by appropriate early treatment by the HEMS. It will be necessary to further compare the ambulance service with the HEMS to evaluate their efficacy in the future.

## Introduction

The Kanagawa Helicopter Emergency Medical Service (HEMS) was started in 2002 and transports various critical cases. These include cases of acute poisoning, and the HEMS contributes to shortening the time to physician contact by hastening transport. The Japanese HEMS is funded by national taxes, therefore its efficacy should be scrutinized. Trauma and vascular disorders associated with HEMS have been studied. However, as the characteristics of the poisoning cases conveyed by the HEMS are not apparent, it has become important to examine the efficacy and safety of HEMS transport in this context. We examined the characteristics of acute poisoning cases conveyed by the Kanagawa HEMS during the study period.

## Method

The Kanagawa HEMS transported 3,814 patients from July 2002 to March 2013 and 131 cases were diagnosed with acute poisoning. We undertook a retrospective descriptive statistical study of these cases.

## Results

### Sex and age

The study included 66 male and 65 female patients; the sex ratio was even and the average age was 49.9 years (standard deviation [SD], ±22 years).

### Emergency services or hospital transfer

In 119 cases (90.8%), the transport requests were from the emergency services in the field, and in 12 cases (9.2%), the hospital transfer request was from a medical institution.

### Number of dispatches

**Figure 1:**
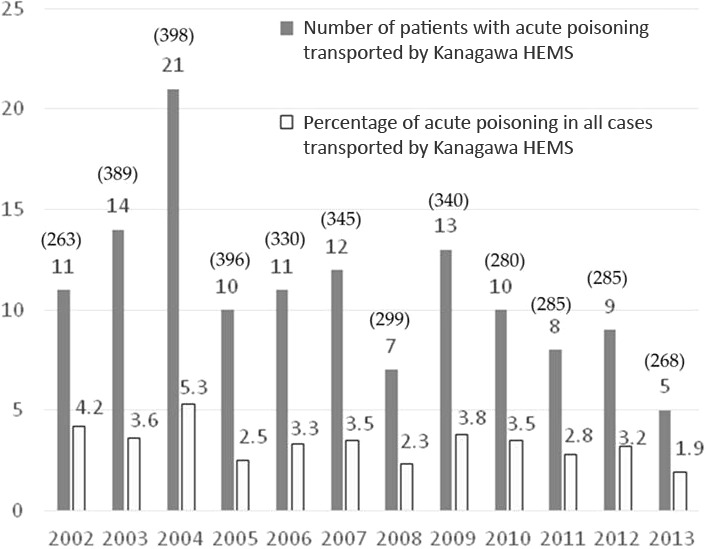
Number of patients with acute poisoning transported by Kanagawa Helicopter Emergency Medical Service (HEMS) from July 2002 to March 2013 and the percentage of poisoning cases relative to all patients. Numbers in parentheses are the number of patients transported by Kanagawa HEMS annually.

The total number of all dispatches and the number of cases of acute poisoning transported by the Kanagawa HEMS for each year, as well as ratios, are shown in Figure 1.

### Main causative drug or poison

**Figure 2:**
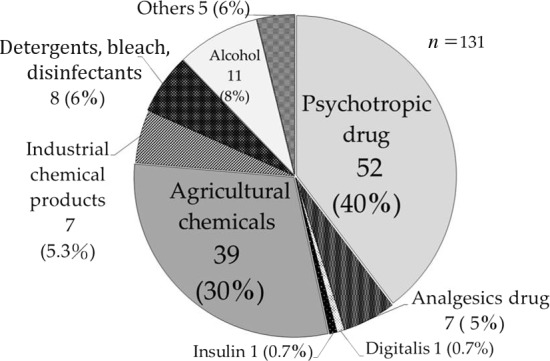
Main causative drugs/agents of poisoning in 131 patients with drug/poisoning intoxication transported by Kanagawa Helicopter Emergency Medical Service from July 2002 to March 2013. Data are shown as number of patients (percentage).

In cases of multiple ingestion, the most active agent determined the priority of treatment. There were 61 cases of poisoning with medical or pharmaceutical products (intended for ingestion or injection), comprising 46.5% of all patients with acute poisoning transported by the HEMS. Of these, 52 cases involved psychotropic drugs (40%), while analgesics, such as non‐steroidal anti‐inflammatory drugs and acetaminophen, accounted for 7 cases (5%). Digitalis and insulin were involved in one case each. Toxins excluding medical agents were 53.4% of the whole; these comprised mainly organic phosphorus and paraquat, a pesticide, in 39 cases (30%), organic solvent or petroleum in 7 cases (5.3%), detergent, bleach or disinfectant in 8 cases (6%), alcohol in 11 cases (8%), and hydrogen sulfide, critical drugs, amphetamine, or an unknown agent in 5 cases (6%) (Fig. 2).

### State of patients on HEMS arrival

Patients’ vital signs were recorded when the HEMS arrived in the field. Mild disturbance of consciousness (patient able to talk) was seen in 37 cases (28.2%), moderate disturbance of consciousness (difficulty in speech) was seen in 28 cases (21.4%), and 66 patients (50.4%) were in a coma. In 24 cases (18.3%) systolic blood pressure was less than 90 mmHg.

With regard to prehospital treatment which was instituted at the field and in flight, 56 cases (42.7%) underwent endotracheal intubation, 130 cases (99.2%) were given transfusion, in over 20 cases (15.2%) a nasogastric tube was inserted, and there were at least 36 cases (27.5%) that received medication. These were mostly adrenalin for cardiac arrest, vasopressors for hypotension, and sedatives to facilitate endotracheal intubation. In addition, treatment with atropine sulfate was often required for bradycardia in organophosphorus pesticide poisoning, as well as antihypertensive agents and sodium bicarbonate in other situations.

### Comparison between medical agents and other toxic causative agents before arrival at hospital

We defined “medical agents” as a group of therapeutic products intended for ingestion or injection in the body. Causative toxic agents that were not medical products and external medical agents were grouped as “other agents”. We compared them with regard to patient situation after arrival of the HEMS and before arrival at the hospital.

With regard to vital signs at HEMS contact, there was no difference in the ratio of number of cases with systolic blood pressure less than 90 mmHg, or in a coma, between the medical agent's group and the other agents group.

These was no difference in the ratio of number of cases in which endotracheal intubation was carried out before arrival at the hospital. Hypotensive and comatose cases were mostly due to psychotropic drug poisoning, and, for cases in which endotracheal intubation was carried out before arrival at the hospital, pesticide poisoning was the most common cause. Death, as a final outcome, was seen in 11 cases in the other agents group, but no fatality was observed in the medical agents group.

### Fatal cases

**Figure 3:**
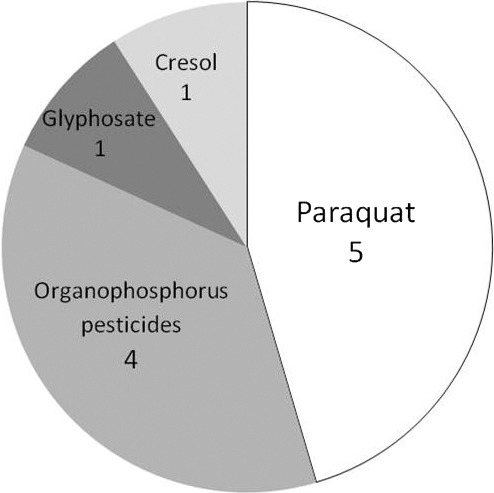
Fatal cases of acute drug intoxication transported by Kanagawa Helicopter Emergency Medical Service from July 2002 to March 2013 (*n* = 11; 8.4%). The most common drug/agent causing death was paraquat.

Death was the final outcome In 11 cases; mortality in patients with acute drug intoxication conveyed by HEMS was 8.4%. The causative agent in the fatal cases was paraquat (five cases), organophosphorus pesticide (four cases), glyphosate (one case), and cresol (one case) (Fig. 3). The fatalities occurring within 24 h included eight patients: five from paraquat and one each from fenitrothion, glyphosate, and cresol.

## Discussion

Forty‐six aircraft were deployed in the HEMS in 38 Japanese prefectures by August 2015. No accidents have occurred and, up to April 2014, the number of flights has exceeded 100,000 since the system was started in 1999. The number of the recent annual dispatch is a tendency to increase. It was 20,515 cases in 2013. The total number of people was 18,851 after we excluded cases for which the service was cancelled after takeoff. There were 3,376 cases of endogenous disease excluding trauma, cardiovascular disease, and cerebrovascular disease. Acute drug intoxication is included in that number, but the data are not fully understood. In addition, the number of acute drug intoxication cases conveyed by aeromedical services has been described by only one institution from the USA.^1^ The Kanagawa HEMS was started in July 2002, and there were 3,814 dispatches and exercises by March 2013; of these, acute drug intoxication was the diagnosis in 131 cases. There was no apparent change in tendency in the ratio or the causative agents from year to year.

Considering all patients with acute drug intoxication that came to our hospital during the same period, the average age was 35.8 years. Psychotropic drug poisoning was the cause in 90.0% of all poisonings (average age 35.4 ± 3.22 years [SD]). Pesticide poisoning, which included many severe cases, was the cause in 4.7% (average age 61.6 ± 14.94 years [SD]). This is in contrast to the average age of patients with acute drug intoxication conveyed by the HEMS, which was higher, at 49.9 years.^2^ Moreover, it was seen that there was relatively more pesticide poisoning (at 30%) among these patients. Poisoning by pesticides is usually more severe and has a greater incidence in agricultural areas that lack medical facilities. Because of greater distances involved and the longer time required for conveyance by ambulance, we assume that more of these cases are conveyed by the HEMS.^3^, ^4^

With respect to vital signs at HEMS contact, 50% of cases were in a coma and 18.3% were in shock (systolic blood pressure ≤90 mmHg). These cases require immediate physician contact and prompt treatment. Of measures carried out before arrival at the hospital, we found that endotracheal intubation was frequently needed and was carried out in nearly half the cases. In addition, early decontamination would be indicated for highly toxic material, and measures for decontamination, such as aspiration of gastric contents or swabbing the body, instituted before arrival at the hospital, might be effective. Medication was provided in at least 30% of cases. Except for cases of cardiac arrest, in most cases in Japan, endotracheal intubation, gastric contents aspiration, and medication can be undertaken only after physician contact. We consider that the HEMS, which could provide these interventions quickly in severely affected patients, would be more effective. It is thought that these that these treatments should be applied appropriately, as patients with acute drug intoxication frequently request HEMS assistance.

Comparing medical agents with other agents, there was no difference between the two groups regarding vital signs and the treatment necessary, but the mortality was significantly higher in the other agents group. Severity at HEMS contact does not appear to be different between the medical agents group and the other agents group, but it seems that if a pesticide is associated as the cause, mortality increases well over the medical agents group. Paraquat, in particular, had a high case fatality rate, and 45.5% of fatal cases reported in two previous studies were caused by paraquat.^5^, ^6^ Seven paraquat poisoning cases were transported by the Kanagawa HEMS, and only two of those survived. The severity index of paraquat poisoning of Sawada *et al*. multiplies the time to start of treatment with blood paraquat concentrations to predict survival.^7^ The HEMS, by hastening initiation of therapy, may theoretically improve the prognosis.

**Figure 4:**
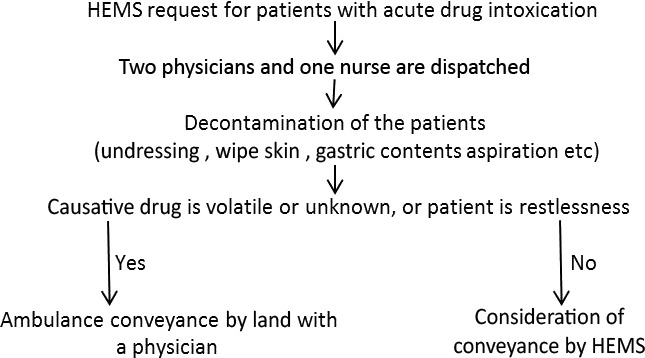
Protocol of the Kanagawa Helicopter Emergency Medical Service (HEMS) following request for assistance for patients with acute drug intoxication. HEMS is dispatched regardless of the causative agent in the field. Decontamination and treatment are provided and conveyance by land or helicopter is determined by the physician with a captain.

Helicopter Emergency Medical Service is requested for many cases of severe acute drug intoxication as speed of conveyance is desired, but safety management is necessary during the flight when the cause is a volatile agent. A volatile drug may pervade a confined helicopter with fumes, and pose danger as a health hazard for pilots, mechanics, physicians, or nurses. Making a pit stop after takeoff is inconvenient (unlike land ambulance), emergency correspondence may be difficult, and there is the risk of an aviation accident. When there is a HEMS request for a case of volatile drug intoxication in the Kanagawa HEMS, we follow the protocol that a physician shares an ambulance and transports the patient by land after decontamination and treatment in the field (Fig. 4). The illustrative cases that follow occurred before the protocol was implemented.

### Case 1

A 60‐year‐old man ingested an unknown amount of fenitrothion emulsion with suicidal intent at home. His family discovered this when he vomited 1 h later, and called for emergency services. At first responder contact, the vital signs were: consciousness clear, respiratory rate 18 breaths/min, pulse not recordable, blood pressure 85/46 mmHg, and SpO_2_ 92% (without supplementary oxygenation). At HEMS contact, vital signs were Glasgow Coma Scale (GCS) E4V4M6, respiratory rate 14 breaths/min, pulse 29 b.p.m., systolic blood pressure 90 mmHg, and SpO_2_ 95% (on oxygen supplementation 10 L/min). We started i.v. fluid 14 min after the HEMS request, and i.v. atropine sulfate for severe bradycardia, an endotracheal tube and a nasogastric tube was inserted, and the helicopter departed with the patient. The patient arrived at the hospital 30 min after the HEMS request, intensive care was provided, and he was eventually discharged without complications. In this case, transfusion initiation and medication for bradycardia and shock could be provided early. In addition, we feel that HEMS was effective in this case, because physician contact was early and decontamination was possible in the early stages by aspiration of gastric contents in the field.^8^

### Case 2

A 50‐year‐old man drank thinner containing acetic acid, n‐butyl, xylene, and isobutanol, in a suicide attempt in the service area of a highway. A passer‐by discovered him when he fell beside a car and called emergency services. At first responder contact, vital signs were: GCS E1V1M4, respiratory rate 20 breaths/min, heart rate 96 b.p.m., systolic blood pressure 110 mmHg, and SpO_2_ 93%. There was profuse frothy sputum in his oral cavity and the odor of thinner on his breath. At HEMS contact, vital signs were GCS E1V1M4, respiratory rate 18 breaths/min, heart rate 91 b.p.m., blood pressure 76/39 mmHg, and SpO_2_ 93% (on oxygen 8 L/min supplementation) and bilateral coarse crackles were audible on chest auscultation. A physician intervened 13 min after the HEMS request and initiated i.v. infusion, performed endotracheal intubation, and aspirated copious frothy sputum from the endotracheal tube. The patient's blood pressure improved to 100/34 mmHg after departure from the site and he arrived at the hospital 32 min after the HEMS request. Intensive care was provided; the patient was extubated on hospital day 3, and was transferred to a psychiatric hospital without physical complications on day 6. Thus, HEMS management was effective in this case because of early endotracheal intubation and infusion for chemical pulmonary edema, which improved the level of consciousness and degree of shock.^9^

## Conclusion

We studied features of acute drug intoxication cases that were conveyed in the Kanagawa HEMS. For severe acute drug intoxication, we regard the early initiation of primary care provided with the HEMS as very important.

## Conflict of Interest

None Declared.
